# Supplementary material for: His108Arg Transthyretin Amyloidosis—Shedding Light on a Distinctively Malignant Variant
Source: J Clin Med. 2024 Dec 23;13(24):7857. doi: 10.3390/jcm13247857 (PMC11728263; doi:10.3390/jcm13247857)
Supplement: Supplementary file 1 [file jcm-13-07857-s001.zip › jcm-3382088-supplementary.pdf]

Supplementary Materials

Table S1- Overview of detected transthyretin mutations during patient and family screening

Sex	Age*	Mutation	Phenotype	DPD scan
Female (Patient 1)	56	His108Arg	Positive	Perugini III
Male	40	His108Arg	Negative	Perugini 0
Male (Patient 2)	56	His108Arg	Positive	Perugini III
Female	59	His108Arg	Negative	Perugini II
Male (Patient 11)	66	His108Arg	Positive	Perugini III
Male	35	His108Arg	Negative	Perugini 0
Male (Patient 12)	60	His108Arg	Positive	Perugini II
Female (Patient 3)	61	His108Arg	Positive	Perugini III
Male (Patient 4)	70	His108Arg	Positive	Perugini III
Male	33	His108Arg	Negative	Perugini 0
Female	40	His108Arg	Negative	Perugini 0
Male (Patient 5)	62	His108Arg	Positive	Perugini III
Female (Patient 7)	60	His108Arg	Positive	Perugini III
Male (Patient 6)	62	His108Arg	Positive	Perugini III
Male (Patient 8)	64	His108Arg	Positive	Perugini III
Female	70	His108Arg	Negative	Perugini I
Female	59	His108Arg	Negative	Perugini 0
Male (Patient 10)	48	His108Arg	Positive	Perugini III
Female (Patient 9)	68	His108Arg	Positive	Perugini III
Female	69	His108Arg	Negative	Perugini 0
Male	68	Thr69Ile	Positive	Perugini III
Male	67	Thr69Ile	Positive	Perugini III
Female	35	Thr69Ile	Negative	Perugini 0
Male	30	Thr69Ile	Negative	Perugini 0
Female	42	Thr69Ile	Negative	Perugini 0
Male	60	Thr69Ile	Positive	Perugini III
Female	72	Ile27Val	Positive	Perugini III
Male	60	Ile127Val	Positive	Perugini III
Female	77	Ile127Val	Positive	Perugini III
Female	42	Ile127Val	Negative	Perugini 0
Female	33	Ile127Val	Negative	Perugini 0
Male	76	Ile127Phe	Positive	Perugini III
Male	59	Ile127Phe	Negative	Perugini 0

Male	80	Val142Ile	Positive	Perugini III
Male	45	Val142Ile	Negative	Perugini 0
Male	45	Val142Ile	Negative	Perugini 0
Male	86	Val30Met	Positive	Perugini III
Male	81	Val30Met	Positive	Perugini III
Male	77	Val30Met	Positive	Perugini III
Female	68	Val114Ala	Positive	Perugini III
Female	75	Val113Leu	Positive	Perugini III
Male	77	Val40Ile	Positive	Perugini III
Male	78	Intron 1: c.69+44G>A)**	Positive	Perugini III
Male	68	Thr80Ala	Positive	Perugini III
Female	87	Cys30Arg	Positive	Perugini III
Male	80	5UTR: c.-61G>A **	Positive	Perugini III
Male	59	Asp94His	Negative	Perugini 0

*In patients with positive cardiac phenotype, age at time of diagnosis is given, while in phenotype negative patients, age at time of screening is shown.
** Mutation in the transthyretin gene of unknown significance. Rows with thick borders indicate that individuals are blood relatives.

Table S2- Overview of neurologic involvement in the patient population with positive cardiac phenotype.

	**Initial,predominant manifestation**	**Previous CTS-surgery**	**Nerve conduction test**	**Clinical presentation**
Patient 1	Cardiac	Unilateral	Bilataral senosmotory PNP of UE and LE	Dysesthesia in both UE
Patient 2	Cardiac	No	No PNP	--
Patient 3	Cardiac	No	No PNP	--
Patient 4	Cardiac	No	Bilataral senosmotory PNP of LE	No significant symptoms
Patient 5	Neurological	No	Bilataral senosmotory PNP of UE and LE	Impaired fine motor skills and gait instability
Patient 6	Cardiac	Bilateral	Bilateral senosmotory PNP of UE and LE	Dysaesthesia of both UE
Patient 7	Cardiac	Bilateral	Bilateral sensomotory PNP of UE < LE	Restless leg symptoms, dysaesthesia of both UE and LE
Patient 8	Cardiac	No	Bilateral sensomotory PNP of UE and LE	Dysesthesia in both UE
Patient 9	Cardiac	Bilateral	Bilateral sensomotory PNP of UE and LE	Dysesthesia in both UE
Patient 10	Cardiac	No	Bilateral, predominantly sensory PNP of LE	No significant symptoms
Patient 11	Cardiac	Unilateral	Bilateral sensomotory PNP of UE and LE	Dysaethesia in left UE
Patient 12	Cardiac	Bilateral	LE-dominant, bilateral sensomotory PNP	No significant symptoms

CTS indicates carpal tunnel syndrome; PNP, polyneuropathy; UE, upper extremities and LE, lower extremities
